# *FTO* rs9939609 and rs17817449 polymorphisms contribute to metabolic syndrome risk by increasing triglyceride and glucose levels

**DOI:** 10.3389/fgene.2025.1659460

**Published:** 2025-11-19

**Authors:** Yongyan Song, Ting Wang, Mi Su, Xue Wang, Youjin Zhang, Jia Pan, Yunhan Wang, Jin Yang

**Affiliations:** 1 Clinical Medical College and Affiliated Hospital of Chengdu University and College of Pharmacy, Chengdu University, Chengdu, Sichuan, China; 2 Functional Science Laboratory, West China School of Basic Medical Sciences and Forensic Medicine, Sichuan University, Chengdu, Sichuan, China; 3 Department of Health Management Center, Clinical Medical College and Affiliated Hospital of Chengdu University, Chengdu, Sichuan, China; 4 Department of Urology Surgery, Clinical Medical College and Affiliated Hospital of Chengdu University, Chengdu, Sichuan, China

**Keywords:** FTO, polymorphism, rs9939609, rs17817449, metabolic syndrome

## Abstract

**Background and Aims:**

The polymorphisms in fat mass and obesity-associated gene (*FTO*) have been implicated in metabolic dysregulation. This study aimed to investigate the associations between the *FTO* rs9939609 and rs17817449 polymorphisms and MetS risk, and to assess whether glucolipid parameters mediate these associations.

**Methods:**

A hospital-based cross-sectional study involving 701 adults was conducted. MetS was diagnosed according to the criteria of the International Diabetes Federation (2005). Clinical data were collected for all participants. Genotyping of rs9939609 and rs17817449 was performed via polymerase chain reaction-restriction fragment length polymorphism. Logistic regression and mediation analysis were used to evaluate genetic associations and mediating effects.

**Results:**

The MetS group showed higher frequencies of rs9939609 A allele (14.01% vs. 6.09%, *P* < 0.001) and rs17817449 G allele (16.94% vs. 12.18%, *P* = 0.012) compared to controls. Rs9939609 AA genotype carriers had the highest MetS risk (OR = 3.58, 95% CI: 1.08–11.88) and exhibited allelic dose-dependent worsening of triglycerides, high-density lipoprotein cholesterol (HDL-C), and fasting blood glucose (FBG) (all *P* < 0.05). Similarly, rs17817449 G allele was linked to elevated triglycerides, reduced HDL-C, higher FBG, and increased systolic blood pressure (all *P* < 0.05). Mediation analysis revealed triglycerides, HDL-C, and FBG as significant mediators for the associations of rs9939609 and rs17817449 with MetS (all *P* < 0.001).

**Conclusion:**

*FTO* rs9939609 and rs17817449 polymorphisms are strongly associated with MetS risk, primarily by increasing triglyceride and glucose levels and decreasing HDL-C. These findings highlight the pivotal role of *FTO* variants in metabolic dysregulation and suggest potential targets for early intervention of MetS.

## Introduction

Metabolic syndrome (MetS) is a cluster of interrelated cardiometabolic risk factors, including abdominal obesity (increased waist circumference, WC), hypertension (elevated systolic/diastolic blood pressure, SBP/DBP), dyslipidemia (high triglycerides; low high-density lipoprotein cholesterol, HDL-C), and impaired glucose metabolism (elevated fasting blood glucose, FBG). Diagnostic criteria vary across guidelines: the NCEP ATP III defines MetS as the presence of ≥3 abnormal components, whereas the International Diabetes Federation (IDF) requires central obesity (based on ethnicity-specific WC cutoffs) plus ≥2 additional components (Third Report of the National Cholesterol Education Program NCEP, 2002; Alberti et al., 2005; Alberti et al., 2006). MetS substantially elevates the risk of cardiovascular disease, type 2 diabetes mellitus (T2DM), metabolic dysfunction-associated steatotic liver disease, and chronic kidney disease (Park et al., 2025; Lai et al., 2025; Alyousef et al., 2025; Li et al., 2023). Its global prevalence ranges from 25% to 33%, with regional variations: 25% in the United States, 30% in Bangladesh, 30.4% in Iran, 31.1% in China, 32.4% in Africa, and 33% in Brazil (Bowo-Ngandji et al., 2023; de Siqueira Valadares et al., 2022; Kalan Farmanfarma et al., 2019; Chowdhury et al., 2018; Falkner and Cossrow, 2014; Yao et al., 2021). The pathogenesis of MetS is multifactorial, involving genetic predisposition, lifestyle factors (e.g., poor diet, physical inactivity), and environmental exposures (e.g., pollutants, endocrine disruptors) (Park et al., 2024; Jeong and Choi, 2024). Recent advances in genomics have identified multiple susceptibility genes and polymorphic variants contributing to MetS susceptibility, highlighting its complex genetic architecture.

The fat mass and obesity-associated gene (*FTO*), which encodes an RNA N6-methyladenosine demethylase, has been implicated in regulating multiple components of MetS. Human studies have demonstrated tissue-specific associations between *FTO* expression and metabolic parameters. Samaras et al. (2010) reported that subcutaneous adipose tissue *FTO* mRNA expression correlated positively with WC and FBG in patients undergoing abdominal surgery. Similarly, Grunnet et al. (2009) found that subcutaneous adipose *FTO* expression in Danish twins showed an inverse relationship with HDL-C levels, suggesting a role in lipid metabolism. Mouse models further reveal the diverse metabolic functions of *FTO* across tissues. Wang et al. (2015) demonstrated that adipose-specific *FTO* knockout mice exhibited enhanced triglyceride clearance and reduced serum triglyceride concentrations through mechanisms involving ANGPTL4 upregulation. In contrast, Luse et al. (2022) showed that smooth muscle cell-specific *FTO* deletion led to significantly reduced SBP and DBP without affecting body weight, revealing a novel role in vascular function regulation. These findings collectively highlight the pleiotropic effects of *FTO* across different tissues in modulating MetS components.

The human *FTO* gene is located on chromosome 16q12.2 and consists of 9 exons interspersed with 8 introns. Two obesity-associated single-nucleotide polymorphisms (SNPs), rs9939609 and rs17817449, reside within intron 1 of *FTO*. The rs9939609 variant, a thymine-to-adenine transversion (g.53786615T>A), has been functionally linked to allele-specific expression differences. Villalobos-Comparán et al. (2008) quantified *FTO* mRNA in subcutaneous adipose tissue from 31 Mexican women, revealing 1.65-fold higher expression in A allele carriers of rs9939609 compared to TT homozygotes. Berulava et al. (2010) extended these findings through transcript quantification in peripheral blood mononuclear cells and skin biopsies, showing A allele carriers exhibited 1.38-fold and 1.31-fold higher *FTO* mRNA levels than TT genotypes, respectively. These expression differences were further corroborated in leukocyte samples by Karra et al. (2013), who observed 1.22-fold increased *FTO* transcription in AA homozygotes compared to T-allele carriers. The rs17817449 variant (g.53779455T>G), positioned approximately 7.2 kb upstream of rs9939609 within the same intronic region, may influence *FTO* expression through cis-regulatory mechanisms. This variant was associated with elevated risks of T2DM and dyslipidemia in a Chinese Han population (Zhang et al., 2023), suggesting its potential role in modulating metabolic phenotypes via *FTO* dysregulation.

This retrospective case-control study aims to evaluate whether *FTO* rs9939609 and rs17817449 polymorphisms are associated with MetS susceptibility, and to elucidate the pathways through which these polymorphisms influence MetS susceptibility.

## Subjects and methods

### Study subjects

This cross-sectional study consecutively enrolled 701 adults (aged ≥18 years) from the Health Management Center at the Clinical Medical College & Affiliated Hospital of Chengdu University between January and December 2024. Eligible participants met the following inclusion criteria: 1) provision of written informed consent; 2) completion of standardized assessments: anthropometry, fasting blood tests (glucose, lipid profile), and blood pressure measurements; 3) availability of complete demographic/lifestyle data (smoking, alcohol drinking). Exclusion criteria included: 1) chronic liver disease such as viral/autoimmune hepatitis, and cirrhosis; 2) endocrine disorders such as Cushing’s syndrome, hypothyroidism, and untreated hyperthyroidism; 3) genetic/metabolic diseases such as hemochromatosis, Wilson’s disease, and glycogen storage disorders; 4) acute conditions such as recent infection, hospitalization, or surgery; 5) current use of glucocorticoids, antipsychotics, or immunomodulators; 6) incomplete biochemical/clinical records. The study was approved by the Ethics Committee of the Clinical Medical College & Affiliated Hospital of Chengdu University.

### MetS diagnosis

MetS was diagnosed according to the IDF criteria (2005) (Alberti et al., 2005), requiring the presence of: 1) WC as an essential component, ≥90 cm for men, and ≥80 cm for women; 2) plus any two of the following four components: triglycerides ≥1.7 mmol/L (150 mg/dL), HDL-C <1.03 mmol/L (40 mg/dL) for men or <1.29 mmol/L (50 mg/dL) for women, SBP/DBP ≥130/85 mmHg or antihypertensive treatment, FBG ≥5.6 mmol/L (100 mg/dL) or T2DM diagnosis. WC was measured at the midpoint between the lower rib and iliac crest. SBP and DBP were measured twice after 5 min rest using a calibrated sphygmomanometer. Triglycerides and HDL-C were analyzed from venous blood samples after 8-h fasting.

### Epidemiological survey and biochemical measurements

All participants answered standardized questionnaires about age, sex, medical history, lifestyle behaviors of smoking and alcohol drinking. Body mass index (BMI) was calculated as weight (in kilograms) divided by height (in meters) squared. Blood samples were tested by laboratory physicians, including measurements of alanine aminotransferase (ALT), aspartate aminotransferase (AST), total cholesterol (TC), low-density lipoprotein cholesterol (LDL-C), apolipoprotein A1 (APOA1), apolipoprotein B (APOB), lipoprotein (a), [Lp(a)], FBG, hemoglobin A1c (HbA1c), uric acid (UA), and hypersensitive C-reactive protein (hsCRP).

### Genomic DNA extraction and genotyping

Genomic DNA was isolated from EDTA-anticoagulated peripheral blood samples using the TIANamp Blood DNA Kit (TIANGEN, Beijing, China) according to the manufacturer’s protocol. DNA concentration and purity were verified by spectrophotometry (NanoDrop2000, Thermo Fisher Scientific), with all samples having A260/A280 ratios between 1.7 and 1.9. The rs9939609 and rs17817449 polymorphisms were genotyped using polymerase chain reaction-restriction fragment length polymorphism analysis. Each 25 μL PCR reaction contained: 1 μL genomic DNA (50–100 ng),12.5 μL 2× Taq PCR Master Mix (containing Taq DNA polymerase, dNTPs, and reaction buffer), 1 μL each of forward and reverse primers (10 μM), and 9.5 μL nuclease-free water. Restriction digestion was performed by incubating 3 μL PCR product with 5 units of appropriate restriction endonuclease. The digested fragments were resolved by electrophoresis on 2.5% agarose gels stained with GoldView nucleic acid dye and visualized under UV transillumination. The primer sequences, expected product sizes, restriction enzymes, and genotype-specific fragment patterns are detailed in Table 1. Supplementary Figure S1 displays the gel electrophoresis patterns of PCR amplification products for the rs9939609 and rs17817449 polymorphisms. Supplementary Figure S2 presents the gel electrophoresis patterns of restriction enzyme digestion products for the rs9939609 polymorphism, while Supplementary Figure S3 shows the corresponding gel electrophoresis patterns of restriction enzyme digestion products for the rs17817449 polymorphism.

**TABLE 1:** Primer sequences, restriction endonucleases, and digested fragments.

Polymorphisms	*FTO* rs9939609	*FTO* rs17817449
Forward primer	5′-CTA​GGT​TCC​TTG​CGA​CTG​CTG​TGA​ACT-3′	5′-GGA​GTC​TCC​CCT​TAA​CTG​GTC-3′
Reverse primer	5′-TTC​AAG​TCA​CAC​TCA​GCC​TCT​CTA​CCA-3′	5′-CAC​AGC​AGG​CAT​TTA​CAA​GCG-3′
Location	Intron	Intron
Product size	215 bp	430 bp
Amplification conditions	95 °C 7 min; 95 °C 30 s, 60 °C 30 s, 72 °C 30 s, 35 cycles; 72 °C 8 min	95 °C 7 min; 95 °C 30 s, 60 °C 30 s, 72 °C 40 s, 35 cycles; 72 °C 10 min
Restriction endonucleases	DdeI C↓TNAG	AlwNI CAGNNN↓CTG
Digested fragments	TT: 215 bp TA: 215 bp, 189 bp and 26 bp AA: 189 bp and 26 bp	GG: 430 bp GT: 430 bp, 357 bp and 73 bp TT: 357 bp and 73 bp

*FTO*, fat mass and obesity-associated gene.

### Statistical analysis

Continuous variables are presented as mean ± standard deviation, and categorical variables as frequencies (percentages). Group comparisons (MetS vs. control) for continuous and categorical variables were performed using independent samples *t*-tests and chi-square tests, respectively. The associations between *FTO* polymorphisms (rs9939609 and rs17817449) and MetS risk were assessed using binary logistic regression, with results reported as odds ratios (ORs) with 95% confidence intervals (CIs). These analyses were adjusted for potential confounding variables, including age, sex, and BMI. Linkage disequilibrium (LD) between the two SNPs (rs9939609 and rs17817449) was assessed by calculating the squared correlation coefficient (r^2^) and Lewontin’s D-prime (D′) in the overall population, as well as separately in the MetS and control groups. LD strength was classified as follows: weak (r^2^ < 0.2), moderate (r^2^ = 0.2–0.8), or strong (r^2^ > 0.8). Mediation analysis was performed using the “Storm Stats” platform (https://zstats.medsta.cn/mediator/), which utilizes the well-established mediation package in R. We employed a simulation-based bootstrap approach with 1,000 iterations to estimate the indirect effects and generate robust 95% CIs. A significant mediation effect was inferred if the 95% CI for the indirect effect did not include zero. The mediation models were specified with the genotypes of rs9939609 and rs17817449 as independent variables. The serum levels of TG, HDL-C, and FBG were evaluated as potential mediators, and the presence of MetS was designated as the dependent variable. All models were adjusted for key covariates, including age, sex, and BMI. All statistical analyses were conducted using R statistical software (version 4.2.0) and EmpowerStats (version 4.2). A two-tailed *P* value <0.05 was considered statistically significant.

## Results

### Characteristics of the study population

The clinical characteristics of the study participants, stratified by the presence of MetS, are presented in Table 2. Compared to the control group, individuals with MetS exhibited a higher prevalence of hypertension (63.52% vs. 17.01%, *P* < 0.001) and T2DM (25.73% vs. 7.11%, *P* < 0.001). Lifestyle behaviors also differed significantly, with a greater proportion of smokers in the MetS group (51.14% vs. 39.09%, *P* = 0.001). Clinically, the MetS group demonstrated markedly elevated SBP and DBP (*P* < 0.001 for both), higher BMI (*P* < 0.001), and increased WC (*P* < 0.001). Metabolic parameters further highlighted significant differences, including elevated triglycerides (*P* < 0.001), reduced HDL-C (*P* < 0.001), and higher FBG (*P* = 0.03). Additionally, the MetS group showed increased levels of ALT (*P* = 0.001), UA (*P* < 0.001) and hsCRP (*P* < 0.001), indicating pronounced systemic inflammation. No significant differences were observed between groups for age, gender distribution, alcohol consumption, or multiple other biochemical parameters including AST, TC, LDL-C, APOA1, APOB, Lp(a), and HbA1c (all *P* > 0.05). These findings collectively underscore the multifaceted metabolic and cardiovascular risk profile associated with MetS.

**TABLE 2:** Clinical characteristics of the study population.

Variables	Control group (*N* = 394)	MetS group (*N* = 307)	*P* value
Demographic variables			
Age, years	53.66 ± 10.54	58.04 ± 11.99	0.20
Male/female	266/128	225/82	0.10
Medical history			
Hypertension, n (%)	67 (17.01%)	195 (63.52%)	<0.001
Diabetes, n (%)	28 (7.11%)	79 (25.73%)	<0.001
Lifestyle behaviors			
Smoking, n (%)	154 (39.09%)	157 (51.14%)	0.001
Alcohol drinking, n (%)	200 (50.76%)	171 (55.70%)	0.19
Clinical variables			
SBP, mmHg	121.42 ± 7.77	144.85 ± 18.19	<0.001
DBP, mmHg	76.16 ± 3.97	90.39 ± 24.36	<0.001
BMI, kg/m^2^	23.69 ± 3.99	27.48 ± 2.26	<0.001
WC, cm	85.37 ± 6.82	91.16 ± 7.12	<0.001
ALT, U/L	28.58 ± 19.28	35.89 ± 21.44	0.001
AST, U/L	26.99 ± 18.28	28.86 ± 12.72	0.76
Triglycerides, mmol/L	1.43 ± 0.23	2.34 ± 0.60	<0.001
TC, mmol/L	4.78 ± 1.23	4.95 ± 0.98	0.054
LDL-C, mmol/L	2.69 ± 0.49	2.84 ± 0.34	0.38
HDL-C, mmol/L	1.66 ± 0.27	1.26 ± 0.22	<0.001
APOA1, g/L	1.45 ± 0.27	1.26 ± 0.3	0.31
APOB, g/L	0.86 ± 0.22	0.83 ± 0.26	0.80
Lp(a), nmol/L	199.78 ± 29.91	228.59 ± 19.02	0.52
FBG, mmol/L	5.51 ± 1.46	6.77 ± 1.82	0.03
HbA1c	5.97 ± 0.43	6.67 ± 0.93	0.10
UA, μmol/L	386.13 ± 85.79	527.95 ± 283.05	<0.001
hsCRP, mg/L	0.79 ± 1.18	3.49 ± 1.62	<0.001

MetS, metabolic syndrome; SBP, systolic blood pressure; DBP, diastolic blood pressure; BMI, body mass index; WC, waist circumference; ALT, alanine aminotransferase; AST, aspartate aminotransferase; TC, total cholesterol; LDL-C, low-density lipoprotein cholesterol; HDL-C, high-density lipoprotein cholesterol; APOA1, apolipoprotein A1; APOB, apolipoprotein B; Lp(a), lipoprotein (a); FBG, fasting blood glucose; HbA1c, hemoglobin A1c; UA, uric acid; hsCRP, hypersensitive C-reactive protein.

### Association of *FTO* rs9939609 and rs17817449 polymorphisms with MetS risk

The analysis of the rs9939609 and rs17817449 polymorphisms revealed significant associations with MetS risk (Table 3). For rs9939609, the MetS group exhibited a markedly higher frequency of the TA genotype (20.85% vs. 10.15%) and AA genotype (3.58% vs. 1.02%) compared to controls (both *P* < 0.001). The combined TA + AA genotypes were also more prevalent in MetS patients (24.43% vs. 11.17%, *P* < 0.001), with the A allele significantly enriched in the MetS cohort (14.01% vs. 6.09%, *P* < 0.001). Similarly, for rs17817449, the GT genotype (28.01% vs. 19.29%) and GG genotype (2.93% vs. 2.54%) were more frequent in MetS patients, with the combined GT + GG genotypes showing a significant difference (30.94% vs. 21.83%, *P* = 0.006). The G allele was also more prevalent in the MetS group (16.94% vs. 12.18%, *P* = 0.012). These findings suggest that both rs9939609 and rs17817449 polymorphisms are strongly associated with an increased risk of MetS, highlighting the potential role of *FTO* gene variants in metabolic dysregulation.

**TABLE 3:** Genotype and allele frequencies of *FTO* rs9939609 and rs17817449 polymorphisms in control and MetS groups.

Polymorphisms	Control group (*N* = 394)	MetS group (*N* = 307)	*P* Value
*FTO* rs9939609			
TT, n (%)	350 (88.83%)	232 (75.57%)	<0.001
AT, n (%)	40 (10.15%)	64 (20.85%)	
AA, n (%)	4 (1.02%)	11 (3.58%)	
AT + AA, n (%)	44 (11.17%)	75 (24.43%)	<0.001
T allele, n (%)	740 (93.91%)	528 (85.99%)	<0.001
A allele, n (%)	48 (6.09%)	86 (14.01%)	
*FTO* rs17817449			
TT, n (%)	308 (78.17%)	212 (69.06%)	0.014
GT, n (%)	76 (19.29%)	86 (28.01%)	
GG, n (%)	10 (2.54%)	9 (2.93%)	
GT + GG, n (%)	86 (21.83%)	95 (30.94%)	0.006
T allele, n (%)	692 (87.82%)	510 (83.06%)	0.012
G allele, n (%)	96 (12.18%)	104 (16.94%)	

*FTO*, fat mass and obesity-associated gene; MetS, metabolic syndrome.

**FIGURE 1:**
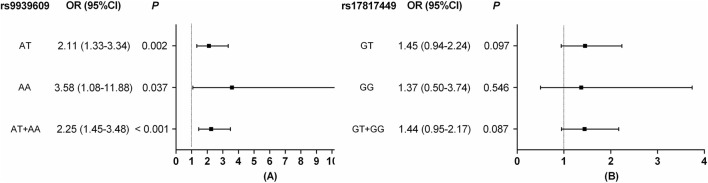
Forest plots of FTO rs9939609 and rs17817449 polymorphisms associated with MetS. **(A)** The reference group was rs9939609 TT genotype; **(B)** the reference group was rs17817449 TT genotype. Both models were adjusted for age, sex, smoking, and drinking.

Figure 1 presents the forest plots of the association between the rs9939609 and rs17817449 polymorphisms and MetS risk based on binary logistic regression analysis with adjustments for age, sex, smoking, and alcohol consumption. For rs9939609 (Panel A), compared to the TT reference genotype, the heterozygous AT genotype showed a significantly increased MetS risk (OR = 2.11, 95% CI: 1.33–3.34). A more pronounced effect was observed in the AA genotype group (OR = 3.58, 95% CI: 1.08–11.88). The combined AT + AA genotypes further confirmed this association (OR = 2.25, 95% CI: 1.45–3.48), underscoring a strong genetic predisposition. In contrast, rs17817449 (Panel B) exhibited a weaker association with MetS risk. The GT genotype (OR = 1.45, *P* = 0.097), GG genotype (OR = 1.37, *P* = 0.55), and combined GT + GG genotypes (OR = 1.44, *P* = 0.087) all failed to reach significance.

### Linkage disequilibrium between rs9939609 and rs17817449

The LD was evaluated between the two *FTO* SNPs, rs9939609 and rs17817449, which are located approximately 7.2 kb apart within intron 1. In the overall study population, the LD measures were r^2^ = 0.24 and D' = 0.61, indicating a moderate level of LD. Stratified analysis revealed stronger LD in the MetS group (r^2^ = 0.37, D' = 0.73) compared to the control group (r^2^ = 0.11, D' = 0.48), suggesting a potential difference in haplotype structure between the two groups. These results confirm that while the two SNPs are in moderate LD, they are not in complete linkage, supporting their independent analysis in this study.

### Association of *FTO* rs9939609 genotypes with MetS components

Analysis of *FTO* rs9939609 genotypes revealed significant associations with key components of MetS (Table 4). In the MetS group, the AA genotype exhibited the most severe metabolic disturbances, with significantly higher triglycerides and lower HDL-C compared to the AT and TT genotypes (all *P* < 0.05). A dose-dependent trend was evident, with stepwise increases in triglycerides and decreases in HDL-C across TT, TA, and AA genotypes (all *P* < 0.05 for trend). Additionally, FBG levels were significantly higher in the AA and AT genotypes compared to the TT genotype (both *P* < 0.001). In the control group, similar but less pronounced patterns were observed. Individuals with the AA or AT genotype had higher triglycerides and lower HDL-C compared to the TT genotype (all *P* < 0.05). No significant differences were noted for WC, SBP, or DBP across genotypes in either group (*P* > 0.05). These findings highlight the graded influence of the rs9939609 variant on dyslipidemia and hyperglycemia, key features of MetS pathogenesis. The AA genotype, in particular, is associated with more severe metabolic abnormalities, underscoring its potential role in MetS susceptibility.

**TABLE 4:** MetS components of the subjects according to *FTO* rs9939609 genotypes.

Genotypes	WC (cm)	SBP (mmHg)	DBP (mmHg)	Triglycerides (mmol/L)	HDL-C (mmol/L)	FBG (mmol/L)
Control group						
TT genotype	85.24 ± 6.32	121.39 ± 16.89	75.99 ± 10.82	1.34 ± 0.52	1.67 ± 0.19	5.49 ± 0.35
AT genotype	86.14 ± 4.85	122.82 ± 14.58	77 ± 10.60	1.74 ± 0.53^a^	1.43 ± 0.08^a^	5.60 ± 0.89
AA genotype	89.29 ± 7.54	110.25 ± 7.77	81.25 ± 10.6	2.14 ± 0.59^a^	1.20 ± 0.01^a^	5.62 ± 1.54
*P*	0.34	0.36	0.57	0.04	<0.001	0.90
MetS group						
TT genotype	90.36 ± 4.61	145.61 ± 19.55	90.67 ± 12.45	2.33 ± 0.52	1.31 ± 0.85	6.41 ± 2.92
AT genotype	91.05 ± 6.37	142.5 ± 21.45	89.09 ± 11.87	2.66 ± 0.62^a^	1.16 ± 0.33^a^	7.74 ± 1.91^a^
AA genotype	92.36 ± 4.64	142.45 ± 19.64	93.14 ± 15.86	3.09 ± 0.79^a^ ^b^	1.01 ± 0.25^a^ ^b^	8.56 ± 4.08^a^
*P*	0.61	0.87	0.95	<0.001	0.001	<0.001

*FTO*, fat mass and obesity-associated gene; MetS, metabolic syndrome; WC, waist circumference; SBP, systolic blood pressure; DBP, diastolic blood pressure; HDL-C, high-density lipoprotein cholesterol; FBG, fasting blood glucose.

^a^ *p* < 0.05 compared with the TT, genotype in control or MetS group.

^b^ *p* < 0.05 compared with the AT, genotype in control or MetS group.

### Association of *FTO* rs17817449 genotypes with MetS components

Analysis of the rs17817449 genotypes revealed distinct associations with MetS components (Table 5). In the MetS group, individuals with the GG genotype showed the most severe metabolic disturbances, exhibiting higher triglyceride levels compared to the GT and TT genotypes (*P* ≤ 0.003), lower HDL-C versus the TT genotype (*P* < 0.05), and elevated FBG relative to GT and TT genotypes (*P* ≤ 0.02). A clear allelic dosage effect was observed for glucolipid parameters, with progressive worsening from TT to GT to GG genotypes (all *P* ≤ 0.01 for trend). The GT genotype displayed intermediate metabolic alterations, with significantly higher triglycerides and lower HDL-C compared to the TT genotype (both *P* < 0.05). In the control group, similar trends emerged but with milder effects. The GG or GT genotype had higher triglycerides than the TT genotype (both *P* < 0.05). The GG genotype had lower HDL-C than the GT or TT genotype (both *P* < 0.05). Notably, SBP was significantly higher in the GG genotype compared to the GT or TT (both *P* < 0.05), suggesting early blood pressure dysregulation. No significant differences were observed for WC or DBP across genotypes in either group (*P* > 0.05). These results underscore the GG genotype’s robust association with dyslipidemia and hyperglycemia, mirroring the *FTO* rs17817449 variant’s impact but with additional links to elevated SBP in controls. The consistency of dose-dependent effects highlights *FTO*’s broader role in metabolic dysregulation.

**TABLE 5:** MetS components of the subjects according to *FTO* rs17817449 genotypes.

Genotypes	WC (cm)	SBP (mmHg)	DBP (mmHg)	Triglycerides (mmol/L)	HDL-C (mmol/L)	FBG (mmol/L)
Control group						
TT genotype	78.98 ± 22.48	120.37 ± 15.79	75.98 ± 10.69	1.36 ± 0.52	1.67 ± 0.43	5.48 ± 1.56
GT genotype	82.51 ± 17.95	123.56 ± 12.69	76.35 ± 9.37	1.63 ± 0.57^a^	1.56 ± 0.29	5.54 ± 1.35
GG genotype	89.78 ± 6.53	136.11 ± 20.83^a^ ^b^	79.44 ± 15.34	1.67 ± 0.32^a^	1.34 ± 0.18^a^ ^b^	5.83 ± 1.03
*P*	0.18	0.01	0.66	<0.001	0.01	0.75
MetS group						
TT genotype	90.72 ± 8.11	142.03 ± 19.24	90.82 ± 12.33	2.22 ± 0.50	1.33 ± 0.36	6.60 ± 1.82
GT genotype	92.14 ± 7.58	141.06 ± 20.63	88.62 ± 11.67	2.57 ± 0.66^a^	1.24 ± 0.31^a^	6.99 ± 1.63
GG genotype	92.78 ± 6.80	145.11 ± 20.41	95.11 ± 16.00	3.16 ± 0.68^a^ ^b^	1.09 ± 0.10^a^	8.47 ± 2.66^a^ ^b^
*P*	0.68	0.85	0.32	<0.001	<0.001	0.007

*FTO*, fat mass and obesity-associated gene; MetS, metabolic syndrome; WC, waist circumference; SBP, systolic blood pressure; DBP, diastolic blood pressure; HDL-C, high-density lipoprotein cholesterol; FBG, fasting blood glucose.

^a^ *p* < 0.05 compared with the TT, genotype in control or MetS group.

^b^ *p* < 0.05 compared with the GT, genotype in control or MetS group.

### Mediation effects of glucolipid parameters on *FTO* rs9939609 and rs17817449 polymorphisms and MetS risk

**FIGURE 2:**
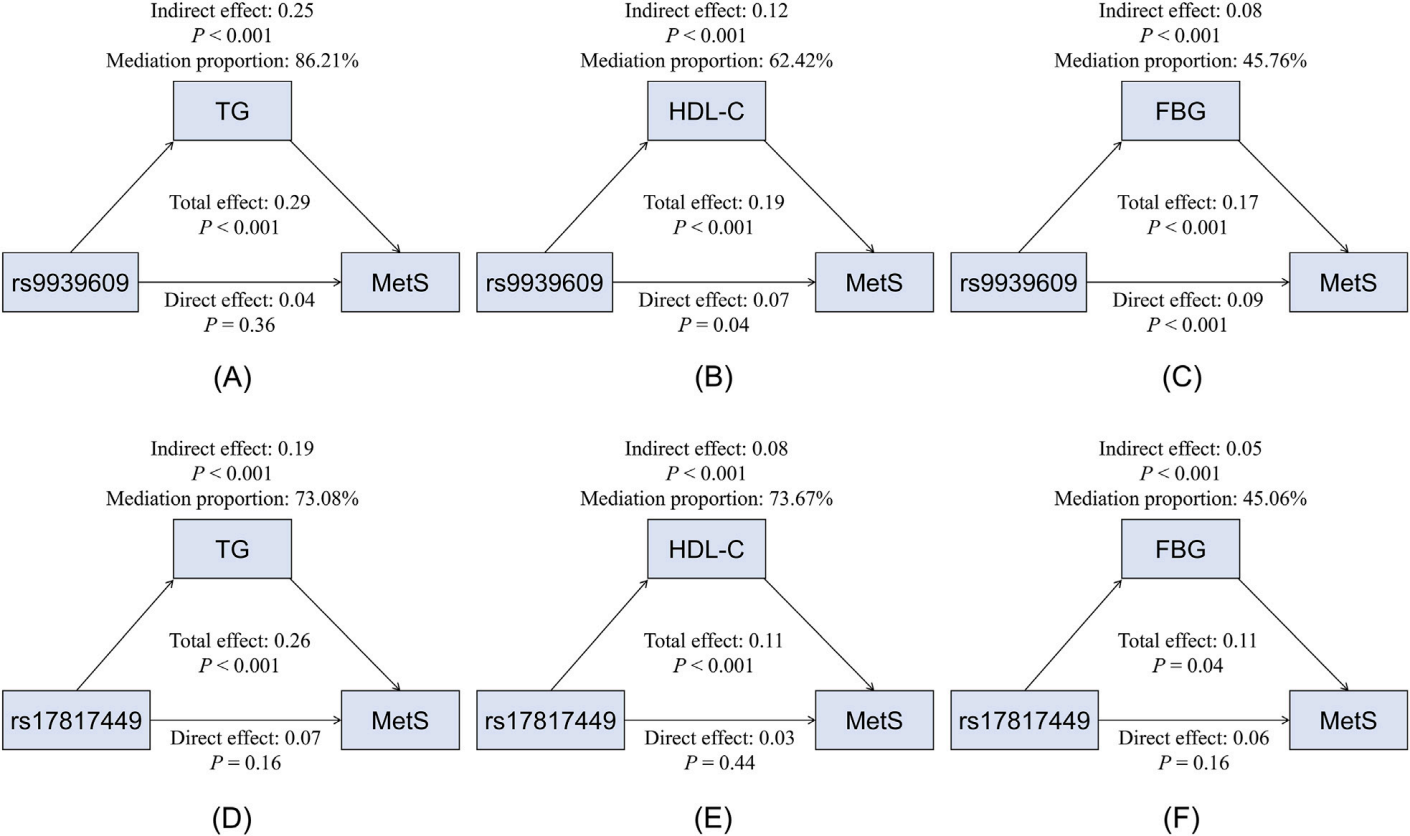
Mediation effects of glucolipid parameters on the association between the rs9939609 and rs17817449 polymorphisms and MetS risk. **(A)** Mediation model for rs9939609 and TG. **(B)** Mediation model for rs9939609 and HDL-C. **(C)** Mediation model for rs9939609 and FBG. **(D)** Mediation model for rs17817449 and TG. **(E)** Mediation model for rs17817449 and HDL-C. **(F)** Mediation model for rs17817449 and FBG. MetS, metabolic syndrome; TG, triglyceride; HDL-C, high-density lipoprotein cholesterol; FBG, fasting blood glucose.

Mediation analysis revealed that triglycerides, HDL-C, and FBG significantly mediated the associations between rs9939609 or rs17817449 and MetS risk (all mediation *P* < 0.001) (Figure 2). Triglycerides exhibited a strong positive mediation effect, accounting for 86.21% (rs9939609) and 73.08% (rs17817449) of the total effect. HDL-C showed a significant negative mediation effect, explaining 62.42% (rs9939609) and 73.67% (rs17817449) of the association. FBG also significantly mediated the relationship positively, though to a lesser extent (45.76% and 45.06%, respectively).

## Discussion

This study provides robust evidence that the FTO polymorphisms rs9939609 and rs17817449 significantly increase MetS risk in a Chinese population, primarily through detrimental effects on glucose and lipid metabolism. Key findings include: 1) The A allele of rs9939609 and G allele of rs17817449 were significantly enriched in MetS patients; 2) Both variants demonstrated allele dose-dependent worsening of MetS components; and 3) Dyslipidemia and hyperglycemia work as the primary pathways linking FTO variants to MetS susceptibility. While large-scale GWAS have primarily established FTO’s link to obesity (Frayling et al., 2007), our results refine and extend this paradigm by demonstrating that its metabolic impact is substantially mediated through glucolipid pathways. Our findings not only align with prior evidence linking FTO to general metabolic dysregulation (Samaras et al., 2010; Grunnet et al., 2009; Wang et al., 2015; Luse et al., 2022), but, more importantly, quantify the substantial mediating role (45%–86%) of these metabolites—a relationship suggested but not rigorously established in previous, smaller-scale studies.

Our findings extend prior research on FTO and metabolic traits while also revealing important distinctions. While earlier large-scale GWAS and meta-analyses have primarily and robustly linked FTO variants to elevated BMI and obesity risk (Frayling et al., 2007; Quan et al., 2015), our mediation analysis demonstrates that a substantial portion of MetS risk is attributable to dyslipidemia and hyperglycemia, partially independent of absolute adiposity measures. Given that these glucolipid abnormalities are not fully explained by adiposity, our data suggest that FTO’s metabolic effects may operate through parallel pathways: one influencing energy balance (appetite/adiposity) and another more directly perturbing hepatic lipid metabolism and insulin signaling. Notably, the rs17817449 G allele showed stronger associations with elevated SBP in controls, an observation that, while not aligning with the primary metabolic profile of FTO, parallels its broader association with cardiovascular risk factors. Although such direct associations with blood pressure are uncommon for FTO variants, isolated reports exist, underscoring the need for further investigation (Sharma and Badaruddoza, 2024). Finally, discrepancies with studies predominantly conducted in European populations (Górczyńska-Kosiorz et al., 2024; Ślęzak et al., 2018) may reflect ethnic-specific genetic architectures or gene-environment interactions, as suggested by trans-ethnic meta-analyses which have noted heterogeneity in FTO effect sizes across ancestries (Wang et al., 2012). Furthermore, the LD analysis revealed a moderate linkage between rs9939609 and rs17817449 in the overall population (r^2^ = 0.24), which was notably stronger in the MetS group (r^2^ = 0.37) than in controls (r^2^ = 0.11). This differential LD pattern may reflect distinct haplotype structures contributing to MetS susceptibility, or could be a consequence of the disease-associated allele distribution.

Our mediation analysis identified triglycerides, HDL-C, and FBG as the significant mediators linking *FTO* polymorphisms to MetS, while BP did not emerge as a primary pathway. This finding aligns with the predominant metabolic function of *FTO*. The gene’s role as an RNA m6A demethylase places it squarely within pathways governing hepatic lipogenesis (Hu et al., 2020; Chen et al., 2018; Tang et al., 2023), adipocyte differentiation (Chen et al., 2022), and insulin signaling (Chen et al., 2022; Zhuang et al., 2019), thereby directly impacting circulating lipid and glucose levels. Conversely, hypertension pathogenesis is largely driven by separate mechanisms, such as renal sodium handling, vascular resistance, and the renin-angiotensin system (Ma et al., 2023), in which *FTO* does not play a well-established central role. This mechanistic divergence is corroborated by our genotype-phenotype analysis (Tables 4 and 5), where both rs9939609 and rs17817449 exhibited clear, dose-dependent effects on triglyceride and FBG levels, but not on SBP or DBP. Therefore, the contribution of these specific *FTO* variants to MetS appears to be channeled predominantly through the dysregulation of glucolipid metabolism, rather than through direct effects on blood pressure.

The mechanisms by which *FTO* rs9939609 and rs17817449 polymorphisms contribute to MetS likely involve epigenetic regulation of metabolic genes and disruption of energy homeostasis, mirroring findings from related obesity-associated *FTO* variants. First, these intronic variants may exert their metabolic effects by modulating *FTO* expression, thereby altering RNA methylation (m6A) patterns of downstream targets critical for lipid and glucose metabolism. Previous studies have consistently shown that the A allele carriers of rs9939609 exhibit elevated *FTO* mRNA levels in subcutaneous adipose tissue (Villalobos-Comparán et al., 2008; Villalobos-Comparán et al., 2017), skin biopsies (Karra et al., 2013), and peripheral blood cells (Berulava et al., 2010). This upregulation disrupts the expression of key metabolic regulators including sterol regulatory element binding protein-1c (Hu et al., 2020; Chen et al., 2018; Tang et al., 2023), peroxisome proliferator-activate receptor-alpha (Wei et al., 2022), peroxisome proliferator-activated receptor-gamma (Chen et al., 2022), peroxisome proliferator-activated receptor gamma coactivator-1 alpha (Zhuang et al., 2019), and carbohydrate responsive element binding protein (Tang et al., 2023). These transcription factors collectively govern critical metabolic processes such as lipogenesis, adipogenesis, and insulin sensitivity. Notably, the pivotal role of FTO in lipid metabolism is supported by large-scale genetic studies that link its risk variants to altered blood lipid profiles, particularly elevated triglycerides, independently of BMI (Fall et al., 2013; Jalili et al., 2021). Second, *FTO* variants may influence appetite regulation and energy intake through ghrelin signaling (Karra et al., 2013). The A allele of rs9939609 has been linked to decreased m6A methylation of ghrelin mRNA, leading to elevated circulating ghrelin levels. This epigenetic dysregulation promotes increased hunger and caloric intake, providing a mechanistic explanation for the allele dose-dependent deterioration in triglycerides, HDL-C, and FBG observed in our study. Together, these mechanisms suggest that FTO-associated MetS risk arises from a combination of direct effects on glucolipid metabolism and indirect effects on feeding behavior. This molecular model directly explains our clinical findings, providing a plausible foundation for the prominent mediating effects of triglycerides, HDL-C, and FBG, and thereby bridging the gap between the FTO genotype and systemic metabolic dysfunction.

The strong associations between *FTO* polymorphisms and MetS risk, particularly through dyslipidemia and hyperglycemia, have important clinical and public health implications. Our findings suggest that genetic screening for *FTO* variants could help identify high-risk individuals before overt metabolic dysfunction develops. Given the allelic dose-dependent worsening of triglycerides, HDL-C, and FBG, carriers of risk genotypes may benefit from early lifestyle interventions or pharmacological strategies targeting lipid and glucose metabolism. Additionally, the high mediation proportions of triglycerides and HDL-C suggest that lipid-lowering therapies might be particularly effective in attenuating genetic risk in this population. These results align with emerging precision medicine approaches for MetS, where genetic profiling could guide risk stratification and tailored prevention strategies.

Several limitations of this study should be acknowledged. First, the cross-sectional design precludes causal inferences regarding the temporal relationship between *FTO* polymorphisms and MetS development. Second, while we adjusted for major confounders, residual confounding from unmeasured factors (e.g., dietary patterns, physical activity intensity, or environmental exposures) may persist. Third, this was a candidate-gene study focusing on two specific *FTO* polymorphisms (rs9939609 and rs17817449) based on our previous research findings (Zhang et al., 2023; Song et al., 2023; Song et al., 2025). A genome-wide association study might reveal additional genetic variants associated with MetS in this population. These limitations highlight the need for longitudinal, multi-ethnic studies incorporating comprehensive omics approaches to validate and extend our findings.

In conclusion, the polymorphisms in the first intron of *FTO* are associated with high risk of MetS, which is strongly modulated by triglycerides, HDL-C and FBG. These findings highlight the necessity for population-specific approaches in genetic risk assessment for MetS.

## Data Availability

The raw data supporting the conclusions of this article will be made available by the authors, without undue reservation.

## References

[B1] Alberti K. G. Zimmet P. Shaw J. IDF Epidemiology Task Force Consensus Group (2005). The metabolic syndrome--a new worldwide definition. Lancet 366 (9491), 1059–1062. 10.1016/S0140-6736(05)67402-8 16182882

[B2] Alberti K. G. Zimmet P. Shaw J. (2006). Metabolic syndrome--a new world-wide definition. A consensus statement from the international diabetes Federation. Diabet. Med. 23 (5), 469–480. 10.1111/j.1464-5491.2006.01858.x 16681555

[B3] Alyousef A. M. Mekawy D. Z. Bashumeel Y. Y. Mohamed S. M. Almigbal T. H. Batais M. A. (2025). The prevalence of metabolic syndrome in patients with non-alcoholic fatty liver disease in primary care clinics at king saud university medical city, Riyadh, Saudi Arabia. Front. Endocrinol. (Lausanne) 16, 1551201. 10.3389/fendo.2025.1551201 40248146 PMC12003115

[B4] Berulava T. Horsthemke B. (2010). The obesity-associated SNPs in intron 1 of the FTO gene affect primary transcript levels. Eur. J. Hum. Genet. 18 (9), 1054–1056. 10.1038/ejhg.2010.71 20512162 PMC2987405

[B5] Bowo-Ngandji A. Kenmoe S. Ebogo-Belobo J. T. Kenfack-Momo R. Takuissu G. R. Kengne-Ndé C. (2023). Prevalence of the metabolic syndrome in African populations: a systematic review and meta-analysis. PLoS One 18 (7), e0289155. 10.1371/journal.pone.0289155 37498832 PMC10374159

[B6] Chen A. Chen X. Cheng S. Shu L. Yan M. Yao L. (2018). FTO promotes SREBP1c maturation and enhances CIDEC transcription during lipid accumulation in HepG2 cells. Biochim. Biophys. Acta Mol. Cell Biol. Lipids 1863 (5), 538–548. 10.1016/j.bbalip.2018.02.003 29486327

[B7] Chen L. S. Zhang M. Chen P. Xiong X. F. Liu P. Q. Wang H. B. (2022). The m(6)A demethylase FTO promotes the osteogenesis of mesenchymal stem cells by downregulating PPARG. Acta Pharmacol. Sin. 43 (5), 1311–1323. 10.1038/s41401-021-00756-8 34462564 PMC9061799

[B8] Chowdhury M. Z. I. Anik A. M. Farhana Z. Bristi P. D. Abu Al Mamun B. M. Uddin M. J. (2018). Prevalence of metabolic syndrome in Bangladesh: a systematic review and meta-analysis of the studies. BMC Public Health 18 (1), 308. 10.1186/s12889-018-5209-z 29499672 PMC5833131

[B9] de Siqueira Valadares L. T. de Souza L. S. B. Salgado Júnior V. A. de Freitas Bonomo L. de Macedo L. R. Silva M. (2022). Prevalence of metabolic syndrome in Brazilian adults in the last 10 years: a systematic review and meta-analysis. BMC Public Health 22 (1), 327. 10.1186/s12889-022-12753-5 35172790 PMC8848905

[B10] Falkner B. Cossrow N. D. (2014). Prevalence of metabolic syndrome and obesity-associated hypertension in the racial ethnic minorities of the United States. Curr. Hypertens. Rep. 16 (7), 449. 10.1007/s11906-014-0449-5 24819559 PMC4083846

[B11] Fall T. Hägg S. Mägi R. Ploner A. Fischer K. Horikoshi M. (2013). The role of adiposity in cardiometabolic traits: a Mendelian randomization analysis. PLoS Med. 10 (6), e1001474. 10.1371/journal.pmed.1001474 23824655 PMC3692470

[B12] Frayling T. M. Timpson N. J. Weedon M. N. Zeggini E. Freathy R. M. Lindgren C. M. (2007). A common variant in the FTO gene is associated with body mass index and predisposes to childhood and adult obesity. Science 316 (5826), 889–894. 10.1126/science.1141634 17434869 PMC2646098

[B13] Górczyńska-Kosiorz S. Lejawa M. Goławski M. Tomaszewska A. Fronczek M. Maksym B. (2024). The impact of haplotypes of the FTO gene, lifestyle, and dietary patterns on BMI and metabolic syndrome in Polish young adult men. Nutrients 16 (11), 1615. 10.3390/nu16111615 38892547 PMC11174437

[B14] Grunnet L. G. Nilsson E. Ling C. Hansen T. Pedersen O. Groop L. (2009). Regulation and function of FTO mRNA expression in human skeletal muscle and subcutaneous adipose tissue. Diabetes 58 (10), 2402–2408. 10.2337/db09-0205 19587359 PMC2750213

[B15] Hu Y. Feng Y. Zhang L. Jia Y. Cai D. Qian S. B. (2020). GR-mediated FTO transactivation induces lipid accumulation in hepatocytes via demethylation of m(6)A on lipogenic mRNAs. RNA Biol. 17 (7), 930–942. 10.1080/15476286.2020.1736868 32116145 PMC7549648

[B16] Jalili V. Mokhtari Z. Rastgoo S. Hajipour A. Bourbour F. Gholamalizadeh M. (2021). The association between FTO rs9939609 polymorphism and serum lipid profile in adult women. Diabetol. Metab. Syndr. 13 (1), 138. 10.1186/s13098-021-00754-0 34801066 PMC8606052

[B17] Jeong S. Choi Y. J. (2024). Investigating the influence of heavy metals and environmental factors on metabolic syndrome risk based on nutrient intake: machine learning analysis of data from the eighth korea national health and nutrition examination survey (KNHANES). Nutrients 16 (5), 724. 10.3390/nu16050724 38474852 PMC10934821

[B18] Kalan Farmanfarma K. Kaykhaei M. A. Adineh H. A. Mohammadi M. Dabiri S. Ansari-Moghaddam A. (2019). Prevalence of metabolic syndrome in Iran: a meta-analysis of 69 studies. Diabetes Metab. Syndr. 13 (1), 792–799. 10.1016/j.dsx.2018.11.055 30641809

[B19] Karra E. O'Daly O. G. Choudhury A. I. Yousseif A. Millership S. Neary M. T. (2013). A link between FTO, ghrelin, and impaired brain food-cue responsivity. J. Clin. Invest 123 (8), 3539–3551. 10.1172/JCI44403 23867619 PMC3726147

[B20] Lai J. Hao M. Huang X. Chen S. Yan D. Li H. (2025). Novel model predicts type 2 diabetes mellitus patients complicated with metabolic syndrome using retrospective dataset from first affiliated hospital of shenzhen university, China. Int. J. Endocrinol. 2025, 9558141. 10.1155/ije/9558141 40313395 PMC12045690

[B21] Li X. Liang Q. Zhong J. Gan L. Zuo L. (2023). The effect of metabolic syndrome and its individual components on renal function: a meta-analysis. J. Clin. Med. 12 (4), 1614. 10.3390/jcm12041614 36836149 PMC9962508

[B22] Luse M. A. Krüger N. Good M. E. Biwer L. A. Serbulea V. Salamon A. (2022). Smooth muscle cell FTO regulates contractile function. Am. J. Physiol. Heart Circ. Physiol. 323 (6), H1212–h1220. 10.1152/ajpheart.00427.2022 36306211 PMC9678421

[B23] Ma J. Li Y. Yang X. Liu K. Zhang X. Zuo X. (2023). Signaling pathways in vascular function and hypertension: molecular mechanisms and therapeutic interventions. Signal Transduct. Target Ther. 8 (1), 168. 10.1038/s41392-023-01430-7 37080965 PMC10119183

[B24] Park S. Kim S. Kim B. Kim D. S. Kim J. Ahn Y. (2024). Multivariate genomic analysis of 5 million people elucidates the genetic architecture of shared components of the metabolic syndrome. Nat. Genet. 56 (11), 2380–2391. 10.1038/s41588-024-01933-1 39349817 PMC11549047

[B25] Park J. H. Jeong I. Ko G. J. Jeong S. Lee H. (2025). Development of a predictive model for metabolic syndrome using noninvasive data and its cardiovascular disease risk assessments: multicohort validation study. J. Med. Internet Res. 27, e67525. 10.2196/67525 40315452 PMC12084770

[B26] Quan L. L. Wang H. Tian Y. Mu X. Zhang Y. Tao K. (2015). Association of fat-mass and obesity-associated gene FTO rs9939609 polymorphism with the risk of obesity among children and adolescents: a meta-analysis. Eur. Rev. Med. Pharmacol. Sci. 19 (4), 614–623. Available online at: https://pubmed.ncbi.nlm.nih.gov/25753879/ . 25753879

[B27] Samaras K. Botelho N. K. Chisholm D. J. Lord R. V. (2010). Subcutaneous and visceral adipose tissue FTO gene expression and adiposity, insulin action, glucose metabolism, and inflammatory adipokines in type 2 diabetes mellitus and in health. Obes. Surg. 20 (1), 108–113. 10.1007/s11695-009-9952-1 19763711

[B28] Sharma T. Badaruddoza B. (2024). Genetic association of FTO gene polymorphisms with obesity and its related phenotypes: a case-control study. J. Cardiovasc Thorac. Res. 16 (2), 102–112. 10.34172/jcvtr.33038 39253342 PMC11380751

[B29] Ślęzak R. Leszczyński P. Warzecha M. Łaczmański Ł. Misiak B. (2018). Assessment of the FTO gene polymorphisms in Male patients with metabolic syndrome. Adv. Clin. Exp. Med. 27 (11), 1581–1585. 10.17219/acem/75676 30091536

[B30] Song Y. Wade H. Zhang B. Xu W. Wu R. Li S. (2023). Polymorphisms of fat mass and obesity-associated gene in the pathogenesis of child and adolescent metabolic syndrome. Nutrients 15 (12), 2643. 10.3390/nu15122643 37375547 PMC10302564

[B31] Song Y. Li S. Liu H. Liu X. Li J. Wang Y. (2025). Higher risk of metabolic syndrome in children and adolescents and polymorphisms in the fat mass and obesity-associated gene: a systematic review and meta-analysis. Pediatr. Res. 10.1038/s41390-025-04020-1 40169741

[B32] Tang Z. Sun C. Yan Y. Niu Z. Li Y. Xu X. (2023). Aberrant elevation of FTO levels promotes liver steatosis by decreasing the m6A methylation and increasing the stability of SREBF1 and ChREBP mRNAs. J. Mol. Cell Biol. 14 (9), mjac061. 10.1093/jmcb/mjac061 36352530 PMC9951264

[B33] Third Report of the National Cholesterol Education Program (NCEP) (2002). Third report of the national cholesterol education program (NCEP) expert panel on detection, evaluation, and treatment of high blood cholesterol in adults (adult treatment panel III) final report. Circulation 106 (25), 3143–3421. 12485966

[B34] Villalobos-Comparán M. Teresa Flores-Dorantes M. Teresa Villarreal-Molina M. Rodríguez-Cruz M. García-Ulloa A. C. Robles L. (2008). The FTO gene is associated with adulthood obesity in the Mexican population. Obes. (Silver Spring) 16 (10), 2296–2301. 10.1038/oby.2008.367 18719664

[B35] Villalobos-Comparán M. Antuna-Puente B. Villarreal-Molina M. T. Canizales-Quinteros S. Velázquez-Cruz R. León-Mimila P. (2017). Interaction between FTO rs9939609 and the native American-origin ABCA1 rs9282541 affects BMI in the admixed Mexican population. BMC Med. Genet. 18 (1), 46. 10.1186/s12881-017-0410-y 28464932 PMC5414298

[B36] Wang H. Dong S. Xu H. Qian J. Yang J. (2012). Genetic variants in FTO associated with metabolic syndrome: a meta- and gene-based analysis. Mol. Biol. Rep. 39 (5), 5691–5698. 10.1007/s11033-011-1377-y 22189543 PMC3381863

[B37] Wang C. Y. Shie S. S. Wen M. S. Hung K. C. Hsieh I. C. Yeh T. S. (2015). Loss of FTO in adipose tissue decreases Angptl4 translation and alters triglyceride metabolism. Sci. Signal 8 (407), ra127. 10.1126/scisignal.aab3357 26671148

[B38] Wei X. Zhang J. Tang M. Wang X. Fan N. Peng Y. (2022). Fat mass and obesity-associated protein promotes liver steatosis by targeting PPARα. Lipids Health Dis. 21 (1), 29. 10.1186/s12944-022-01640-y 35282837 PMC8918283

[B39] Yao F. Bo Y. Zhao L. Li Y. Ju L. Fang H. (2021). Prevalence and influencing factors of metabolic syndrome among adults in China from 2015 to 2017. Nutrients 13 (12), 4475. 10.3390/nu13124475 34960027 PMC8705649

[B40] Zhang Y. Chen L. Zhu J. Liu H. Xu L. Wu Y. (2023). Minor alleles of FTO rs9939609 and rs17817449 polymorphisms confer a higher risk of type 2 diabetes mellitus and dyslipidemia, but not coronary artery disease in a Chinese Han population. Front. Endocrinol. (Lausanne) 14, 1249070. 10.3389/fendo.2023.1249070 38161971 PMC10754952

[B41] Zhuang C. Zhuang C. Luo X. Huang X. Yao L. Li J. (2019). N6-methyladenosine demethylase FTO suppresses clear cell renal cell carcinoma through a novel FTO-PGC-1α signalling axis. J. Cell Mol. Med. 23 (3), 2163–2173. 10.1111/jcmm.14128 30648791 PMC6378205

