# Supplementary material for: FTO rs9939609 and rs17817449 polymorphisms contribute to metabolic syndrome risk by increasing triglyceride and glucose levels
Source: Front Genet. 2025 Nov 19;16:1659460. doi: 10.3389/fgene.2025.1659460 (PMC12671892; doi:10.3389/fgene.2025.1659460)
Supplement: Supplementary file 1 [file DataSheet1.docx]

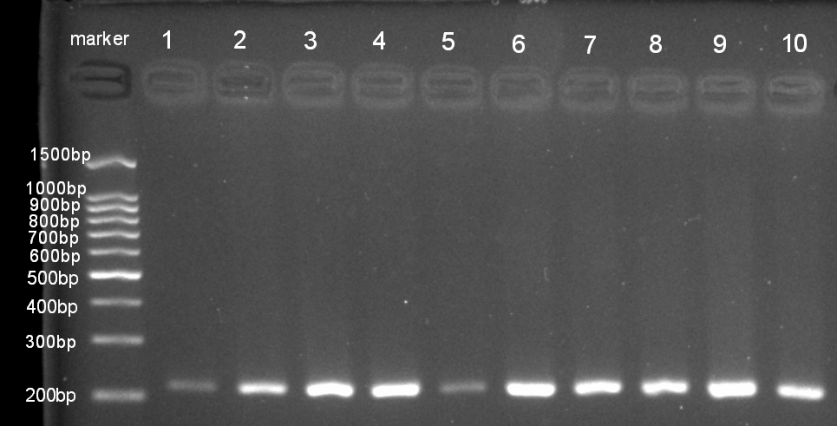

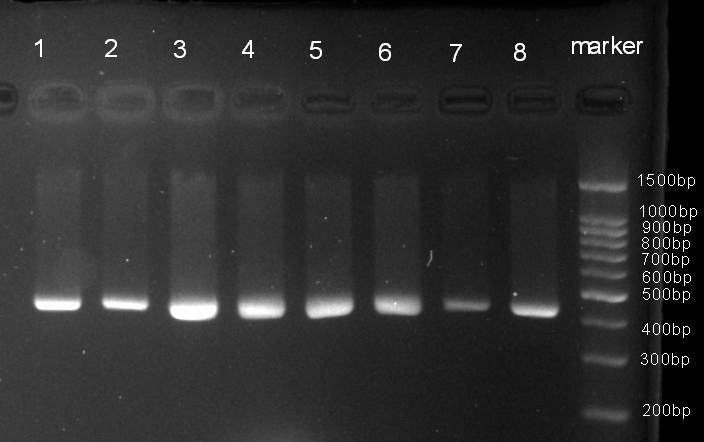


(A) (B)

**Supplementary Figure S1.** Gel electrophoresis map of the PCR amplification products

(A) PCR amplification products for the *FTO* rs9939609 polymorphism (215 bp); (B) PCR amplification products for the *FTO* rs17817449 polymorphism (430 bp).


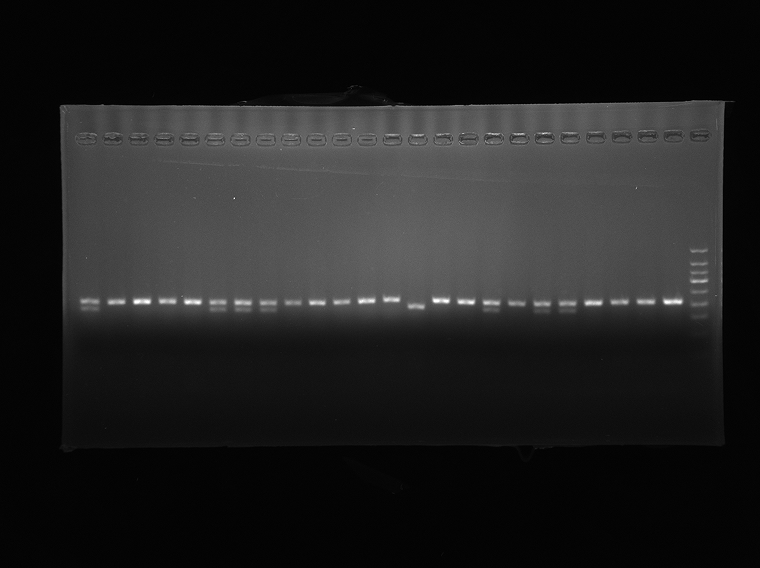


1 2 3 4 5 6 7 8 9 10 11 12 13 14 15 16 17 18 19 20 21 22 23 24 M

215 bp

189 bp

300 bp

150 bp

500 bp

200 bp

400 bp

**Supplementary Figure S2.** Gel electrophoresis map of the *FTO* rs9939609 polymorphism genotyped by PCR-RFLP method.

Lanes 2, 3, 4, 5, 9, 10, 11, 12, 13, 15, 16, 18, 21, 22, 23 and 24: TT genotype; Lanes 1, 6, 7, 8,17, 19 and 20: TA genotype; Lane 14: AA genotype.


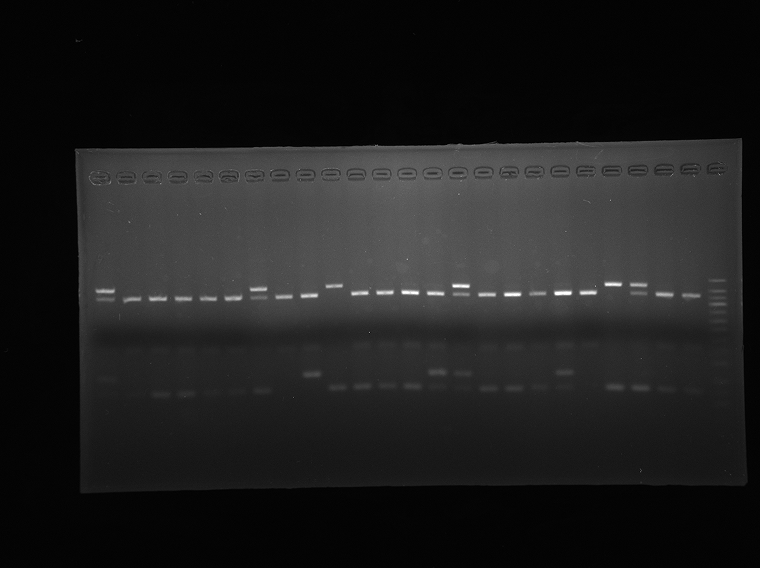


1 2 3 4 5 6 7 8 9 10 11 12 13 14 15 16 17 18 19 20 21 22 23 24 M

430 bp

357 bp

200 bp

300 bp

400 bp

500 bp

100 bp

50 bp

73 bp

**Supplementary Figure S3.** Gel electrophoresis map of the *FTO* rs17817449 polymorphism genotyped by PCR-RFLP method.

Lanes 2-6, 8, 9, 11-14, 16-20, 23 and 24: TT genotype; Lanes 1, 7, 15 and 22: TG genotype; Lanes 10 and 21: GG genotype.
